# Distribution of zoonotic variegated squirrel bornavirus 1 in naturally infected variegated and Prevost’s squirrels

**DOI:** 10.1038/s41598-019-47767-4

**Published:** 2019-08-06

**Authors:** Jana Petzold, Judith M. A. van den Brand, Daniel Nobach, Bernd Hoffmann, Donata Hoffmann, Christine Fast, Chantal B. E. M. Reusken, Peter R. W. A. van Run, Kore Schlottau, Martin Beer, Christiane Herden

**Affiliations:** 1 Institute of Veterinary Pathology, Justus Liebig University, Giessen, Germany; 2 Department of Viroscience, Erasmus Medical Center, Rotterdam, The Netherlands; 3 Institute of Diagnostic Virology, Friedrich-Loeffler-Institut, Greifswald, Germany; 4 National Institute for Public Health and the Environment, Bilthoven, The Netherlands; 5 Present Address: Department of Pathobiology, Utrecht University, Utrecht, The Netherlands

**Keywords:** Central nervous system infections, Viral infection

## Abstract

Recently, the zoonotic capacity of the newly discovered variegated squirrel bornavirus 1 (VSBV-1) was confirmed in humans with a lethal encephalitis. Transmission to humans occurred by variegated and Prevost’s squirrels as presumed reservoir hosts but possible ways of virus shedding and the route of infection still need to be elucidated. Thus, the tissue distribution of VSBV-1 antigen and RNA was investigated in detail via immunohistochemistry (IHC) in six variegated and eight Prevost’s squirrels and by *in situ* hybridisation (ISH) in one Prevost’s squirrel, respectively. VSBV-1 antigen and RNA positive cells were most numerous in the nervous system and were also found in nearly all tissues and different cell types indicating a broad organ and cell tropism of VSBV-1. Presence of VSBV-1 in several organs might indicate potential virus shedding via various routes and implies the risk of intra- and interspecies transmission, respectively.

## Introduction

New zoonotic infections are most commonly emerging from RNA viruses, about half of them cause neurologic diseases and about 80% are zoonotic^1,2^. For bornaviruses, belonging to non-segmented, negative-sense single-stranded RNA-viruses within the order *Mononegavirales*, only the mammalian Borna disease virus 1 (BoDV-1; species *Mammalian 1 orthobornavirus*, family *Bornaviridae*^3^) had been known as cause of a lethal neurological disorder called Borna disease in accidental hosts such as horse and sheep for a long time^4^. Recently, there is evidence that BoDV-1 can cause a comparable clinical lethal manifestation in humans^5,6^. The bicolored white-toothed shrew (*Crocidura leucodon*) represents the only known natural reservoir for BoDV-1 in endemic areas (e.g. Bavaria, Germany) comprising a disseminated tissue distribution of the virus combined with a clinically inapparent and persistent course of infection^7–11^. In 2015, the novel zoonotic variegated squirrel bornavirus 1 (VSBV-1; species *Mammalian 2 orthobornavirus*, family *Bornaviridae*^3^) was detected in three variegated squirrel breeders suffering from a fatal meningoencephalitis and in one contact squirrel (*Sciurus variegatoides*)^12^. Additionally, VSBV-1 was detected in brain tissue of a zoo worker with a fatal limbic encephalitis^13^. After its discovery in Germany, further investigations in several squirrel holdings revealed the presence of VSBV-1 infection in squirrels in the Netherlands and in Croatia as well as in additional *Sciuridae* species including Prevost’s squirrels (*Callosciurus prevostii*), *C*. *finlaysonii*, and *Tamiops swinhoei* within the subfamily *Callosciurinae* and *Sciurus granatensis* within the subfamily *Sciurinae*^14,15^. In the case of the VSBV-1-infected zoo worker, detection of VSBV-1 RNA was restricted to the brain without any detection of viral RNA in peripheral organs available for investigation as known for BoDV-1- infected horses^13^. In contrast, in the naturally infected squirrels virus VSBV-1 RNA was detected in many organs with highest viral RNA loads in the central nervous system (CNS), heart, lung, kidney, oropharyngeal swabs, skin and the urinary bladder^12,14,15^. This likewise dissemination of virus genome in species of the *Callosciurinae* and *Sciurinae* subfamily might identify these species as reservoir hosts capable of shedding infectious virus via several routes comparable to the BoDV-1- infected bicolored white-toothed shrew. It has already been reported that in reservoir-bound infections natural reservoir hosts seem to tolerate the infection, do not show any or only mild pathologic lesions and clinical signs with a typically asymptomatic course of infection^16–18^. However, so far there is limited knowledge on potential shedding pathways, infection routes, tissue distribution and cell tropism of VSBV-1. The scenario observed in the naturally infected squirrels and human contact cases closely resembles the situation for natural BoDV-1 infection in the reservoir, *C*. *leucodon*, and the accidental hosts, horse and sheep. The latter typically develop a non-suppurative meningoencephalitis with a strict neurotropism and persistent infection^4,19^. Similarities in virus tissue distribution and clinical outcome in BoDV-1-infection and VSBV-1-infection in the different (possible) reservoir and accidental host species, respectively, enhance the need for a detailed morphological characterization of VSBV-1-antigen and RNA distribution in the different host systems. This allows to illuminate potential ways of virus shedding by secretions and excretions as prerequisite for an evidence-based assessment of the risk for VSBV-1 transmission to humans and other animal species.

## Results

### Gross findings

One Prevost’s squirrel (P#1) showed a focal adhesion of one lung lobe to the pleura, a pale liver (due to exsanguination), a bilateral enlarged uterus with cysts and a thickened uterine wall and a melanosis of the meninges. Prevost’s squirrel P#2 depicted a slightly lobular pattern of the liver and enlarged tonsils. Three animals (P#3, P#4, P#5) were in a poor to moderate level of nutrition. Additionally, on Prevost’s squirrel P#4 one flea was documented. The Prevost’s squirrel P#5 that died for an unknown reason, depicted a haemothorax and multiple muscular haemorrhages indicating a traumatic origin. Prevost’s squirrel P#8 showed a hepatic neoplasia. No obvious gross lesions were seen in the variegated squirrels.

### Microscopic findings

#### Histology

Main histopathologic changes suspected of being virus-induced and related virus antigen distribution are depicted in Table 1.

**Table 1:** Correlation between histopathologic changes suspected of being virus-induced and related virus antigen distribution.

Organ	Histopathology			Immunohistochemistry (IHC)		
	Score		Character of lesions	Score		Cells positive for VSBV-1 antigen
Brain	+	(6/14)*	Mononuclear meningoencephalitis	− + +(+) ++ +++	(1/14) (5/14) (1/14) (4/14) (3/14)	Neurons, glial cells, Purkinje cells, granule cells
	+	(4/14)	Encephalitis with mononuclear perivascular infiltrates			
	+	(2/14)	Mononuclear meningitis			
	+	(1/14)	Focal malacia with neuronopahgia			
	+	(12/14)	Intranuclear eosinophilic Joest-Degen inclusion bodies			
	+	(10/14)	Satellitosis and gliosis			
Spinal cord	+	(1/12)	Mononuclear myelitis	(+) ++ +++	(1/12) (6/12) (5/12)	Neurons, glial cells, ependymal cells
Trigeminal ganglion	+	(2/11)	Mononuclear ganglioneuritis	− + ++ +++	(1/11) (6/11) (3/11) (1/11)	Neurons, glial cells
Peripheral nerves	+	(2/13)	Mononuclear perineural infiltrates	(+) + ++ ++(+) +++	(2/13) (3/13) (5/13) (1/13) (2/13)	Nerve fibres, peri- and endoneurium
Lung	+ ++	(3/14) (2/14)	BALT-hyperplasia	− (+) + ++	(3/14) (1/14) (9/14) (1/14)	Bronchial epithelium, alveolar macrophages, type 2 pneumocytes, BALT, connective tissue, smooth muscle cells
Kidney	+ ++	(7/14) (2/14)	Non-suppurative interstitial nephritis	− + ++ +++	(5/14) (3/14) (5/14) (1/14)	Tubular epithelial cells, Bowman’s capsule of glomeruli
Tonsil	+	(3/9)	Lymphoid hyperplasia	− + ++	(3/9) (5/9) (1/9)	Cells resembling lymphocytes, interstitial cells, epithelium
Spleen	+	(4/14)	Lymphoid hyperplasia	− + ++	(9/14) (3/14) (2/14)	Cells resembling lymphocytes, interstitial cells
Lymph nodes	+	(5/9)	Lymphoid hyperplasia	− +	(5/8) (3/8)	Cells resembling lymphocytes, interstitial cells

Histopathologic lesions, scored for severity: ++ = moderate; + = mild.

IHC: +++ = high no. of positive cells; ++ = moderate no. of positive cells; + = low no. of positive cells; (+) = questionable;

− = no positive cells.

*x/x = no. of organs/no. of available organs from 14 variegated and Prevost’s squirrels.

**Figure 1:**
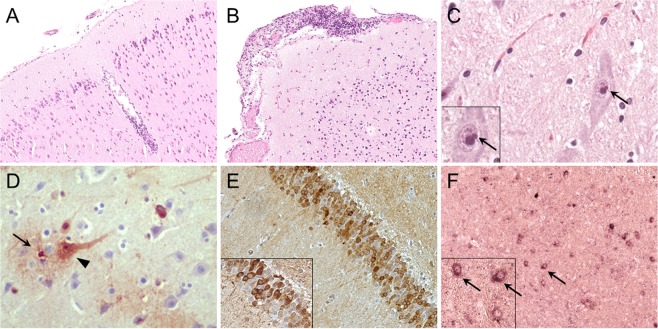
Histology (H&E), immunohistochemistry (IHC) and *in situ* hybridisation (ISH) of brain tissue of variegated squirrel bornavirus 1 (VSBV-1) infected variegated and Prevost’s squirrels. (**A**) Cortex cerebri of Prevost’s squirrel P#7. Mild non-purulent perivascular encephalitis; H&E, original magnification x100. (**B**) Brainstem of variegated squirrel V#1. Mild non-purulent meningitis; H&E, original magnification x100. (**C**) Brainstem of variegated squirrel V#6. Neuron with eosinophilic intranuclear Joest-Degen inclusion body (insert, arrow); H&E, original magnification x400. (**D**) Midbrain of variegated squirrel V#3. Demonstration of VSBV-1 P in neurons (arrowhead) and astrocytes (arrow); IHC, original magnification x400. (**E**) Hippocampus of Prevost’s squirrel P#8. Demonstration of VSBV-1 P in hippocampal neurons; IHC, original magnification x200, magnification of insert x400. (**F**) Cerebrum of Prevost’s squirrel P#8. Demonstration of VSBV-1 genomic RNA in cytoplasm of neurons (arrow); ISH, original magnification x100, magnification of insert x400.

In the **nervous system**, most animals (12/14) revealed mild inflammatory alterations of the brain. 6/14 animals (V#1, V#2, V#5, P#2, P#4, P#8) showed a mild mononuclear meningoencephalitis, 4/14 animals (V#3, V#4, P#3, P#7) showed an encephalitis with mild mononuclear perivascular infiltrates (Fig. 1A) and additionally 2/14 animals (P#1, P#6) had a mild mononuclear meningitis (Fig. 1B). Infiltrates were often accompanied by a mild satellitosis and gliosis (V#1, V#2, V#5, V#6, P#1–3, P#6–8). One squirrel (P#3) also showed focal malacia with neuronophagia. In animal P#5 few mononuclear infiltrates were suspected in the brain but histologic evaluation was limited due to freezing artefacts. In animal V#4 a mild mononuclear myelitis was present. Additionally, two animals (V#1, V#2) showed a mild mononuclear trigeminal ganglioneuritis. Eosinophilic Joest-Degen inclusion bodies that have already been reported in nervous tissue of infected variegated and Prevost’s squirrels were detected in 12/14 animals (Fig. 1C)^15^. Meningeal melanosis was found in 6/14 squirrels (V#2, V#3, P#1, P#3, P#4, and P#7). Two animals (V#1, V#5) showed mild mononuclear perineural infiltrates in peripheral nerve fibres.

In the **respiratory system**, in 2/14 animals (P#3, P#8) a mild purulent rhinitis was present. In the lungs of 2/14 animals (V#1, V#3) a mild often subserosal (pyo)granulomatous bronchopneumonia/bronchitis was found, whereas a BALT-hyperplasia was detected in 5/14 squirrels (V#1–3, V#5 and P#4). Additionally, 2/14 animals showed focal hemorrhage in the lung (P#4, P#8).

Regarding the **digestive system**, intestinal cestoda infection was found in two squirrels (V#6 and P#3). The liver of different animals showed variable lesions, including a well-differentiated hepatocellular carcinoma (P#8), a focal granuloma (P#4) and microabscesses (V#6). In 2/14 (V#2, V#3) animals a mild vacuolation of hepatocytes was present consistent with hepatic lipidosis.

In the **urogenital system**, a mild to moderate non-suppurative interstitial nephritis was present in 9/14 animals (V#1, V#3, V#4, P#1-3, P#6-8), one squirrel exhibited a small infarct scar on the kidney (V#1), single degenerated tubules (P#8) and a uterine leiomyoma (P#3).

Regarding the **lymphoid system**, V#5 exhibited a pyogranulomatous lymphadenitis of the mesenteric lymph node with adjacent purulent steatitis. 9/14 animals showed a lymphoid hyperplasia of the tonsils, spleen and mesenteric lymph nodes.

#### Demonstration of VSBV-1 phosphoprotein (VSBV-1 P) by immunohistochemistry

In general, VSBV-1 antigen was found widespread by IHC in nearly all organ systems in 6/6 naturally infected variegated and 7/8 Prevost’s squirrels. Detection of VSBV-1-antigen failed in one Prevost’s squirrel P#5, that died of unknown reason and was kept frozen, although VSBV-1 RNA was detected by PCR in this animal. In all other 13 animals, virus antigen was, albeit in a different number of positive cells, demonstrated in every organ, besides liver and skeletal muscle. Virus antigen was present in the nucleus and/or in the cytoplasm in cells of neuroectodermal, epithelial and to a lesser extent of mesenchymal origin. A comparison between IHC results and related main histopathologic changes is given in Table 1. Distribution of virus antigen within the examined tissues and cell types are summarised in Table 2. A detailed overview of IHC results for all tissues examined in each animal is given in Table 3 for the variegated and in Table 4 for the Prevost’s squirrels. A comparison of detection of virus antigen and viral RNA by RT-qPCR in previous studies^15^ can be found in Supplementary Tables S1 and S2. Antigen was not detected in the negative control Prevost’s squirrel, data of this squirrel are not depicted in the tables.

**Table 2:** Overview of the cell types demonstrating VSBV-1 antigen by IHC in tissues from infected squirrels.

	6 Variegated squirrels	8 Prevost’s squirrels	Cells positive for VSBV-1 antigen
**Nervous System**			
Central Nervous System	6/6*	7/8*	Neurons, glial cells, Purkinje cells, granule cells, ependymal cells
Peripheral Nervous System	6/6	7/7	Nerve fibres, peri- and endoneurium, neurons of ganglia
**Respiratory System**			
Nose	5/6	5/7	Olfactory and respiratory epithelium, glands, nerve cells, connective tissue
Lung	6/6	5/8	Bronchial epithelium, alveolar walls, alveolar macrophages, type 2 pneumocytes, BALT, connective tissue, smooth muscle cells
**Digestive System**			
Intestine	6/6	6/8	Goblet cells, epithelium, connective tissue and myocytes of lamina propria and muscularis mucosae, tela submucosa, tunica muscularis, ganglia
Liver	0/6	0/8	None
Salivary gland	1/5	5/7	Glandular cells, nerves, vessels
**Urogenital System**			
Kidney	4/6	5/8	(Mainly distal) tubular epithelium, Bowman’s capsule of glomeruli
Urinary Bladder	2/4	6/7	Epithelium, muscularis, glands, nerve cells
Ovary/Uterus	2/2	2/3	Connective tissue, muscle cells, uterine glands
Testes/Penis	3/4	2/5	Connective tissue, muscle cells, urethral epithelium, spermatozoa (and precursor cells)
**Other organs**			
Skin	6/6	7/8	Sebaceous glands, hair follicles, epithelium, sweat glands, nerve cells
Lacrimal gland	3/5	1/1	Glandular cells, connective tissue, nerves, vessels
**Lymphoid organs**			
Tonsil	1/2	5/7	Small round cells with minimal chromatin and a basophilic nucleus, oval to round cells with abundant chromatin and a large nucleus, interstitial cells, epithelium
Spleen	1/6	4/8	Small round cells with minimal chromatin and a basophilic nucleus, oval to round cells with abundant chromatin and a large nucleus, interstitial cells
Lymph nodes	0/4	2/3	Small round cells with minimal chromatin and a basophilic nucleus, oval to round cells with abundant chromatin and a large nucleus, interstitial cells

*x/x = no. of positive organs/no. of available organs from variegated or Prevost’s squirrels.

**Table 3:** Virus distribution by IHC in tissues from VSBV-1-infected variegated squirrels.

	V#1	V#2	V#3	V#4	V#5	V#6
	IHC	IHC	IHC	IHC	IHC	IHC
Brain	++	+	++	+(+)	++	++
Spinal cord	++	+++	+++	++	++	+++
Peripheral nerves	++	++	++(+)	++	++	+++
Nose	++	+	+++	++	−	+++
Trachea	+	+	+	n/a	+	++
Lung	+	+	+	+	+	++
Tongue	n/a	n/a	n/a	n/a	n/a	n/a
Oesophagus	++	+	++	+	+	+
Stomach	+	+	n/a	n/a	n/a	n/a
Small intestine	−	+	+	n/a	−	+
Large intestine	+	+(+)	+(+)	+	++	−
Liver	−	−	−	−	−	−
Pancreas	+	+	++	++	+	++
Kidney	+	−	+	+++	−	++
Urinary bladder	++	n/a	++	−	−	n/a
Ovary	n/a	n/a	n/a	n/a	+	n/a
Uterus	n/a	+	n/a	n/a	−	n/a
Testis	−	n/a	−	+	n/a	−
Penis	−	n/a	+	−	n/a	+
Skin	+	+	+	++	+	+
Eye	+	+	+	n/a	+	++
Lacrimal gland	−	+	n/a	+	−	+
Salivary gland	−	−	−	n/a	−	+
Adrenal gland	n/a	n/a	n/a	n/a	n/a	++
Lymph nodes	−	n/a	−	−	−	n/a
Tonsil	n/a	n/a	n/a	+	−	n/a
Spleen	−	−	−	−	−	+
Bone marrow	n/a	n/a	n/a	n/a	n/a	n/a
Heart	+	+	+	+	−	+
Skeletal muscle	−	−	−	−	−	−

+++ = high no. of positive cells; ++ = moderate no. of positive cells; + = low no. of positive cells; (+) = questionable;

− = no positive cells; n/a = not available.

**Table 4:** Virus distribution by IHC in tissues from eight VSBV-1-infected Prevost’s squirrels and by ISH in one Prevost’s squirrel.

	P#1	P#2	P#3	P#4	P#5	P#6	P#7	P#8		
	IHC	IHC	IHC	IHC	IHC	IHC	IHC	IHC	ISH, mRNA	ISH, gRNA
Brain	+	+	+	+	−	+++	+++	+++	++	+
Spinal cord	++	++	n/a	(+)	n/a	++	+++	+++	+	(+)
Peripheral nerves	+	+	(+)	(+)	n/a	++	+	+++	−	−
Nose	−	+++	+	−	n/a	++	++	+	−	−
Trachea	+	++	−	−	−	+	+	+	−	−
Lung	−	+	(+)	−	−	+	+	+	+	−
Tongue	+	+	+	+	−	n/a	n/a	n/a	n/a	n/a
Oesophagus	n/a	n/a	+	−	−	+	+	+	−	−
Stomach	++	++	+	−	−	n/a	n/a	n/a	n/a	n/a
Small intestine	+	++	+	+	−	++	+	+	+	−
Large intestine	++	+	+	−	−	+	+++	++	+	+
Liver	−	−	−	−	−	−	−	−	−	−
Pancreas	+++	++	+	+	−	++	+	+++	++	++
Kidney	−	++	−	+	−	++	++	++	+	−
Urinary bladder	+	++	+	+	-	n/a	+	++	+	−
Ovary	−	n/a	−	n/a	n/a	n/a	n/a	++	−	−
Uterus	+	n/a	−	n/a	n/a	n/a	n/a	++	+	+
Testis	n/a	+	n/a	−	−	+	−	n/a	n/a	n/a
Penis	n/a	−	n/a	n/a	n/a	+	−	n/a	n/a	n/a
Skin	+	+	+	+	−	++	++	++	+	+
Eye	+	+	+	+	n/a	++	+++	++	−	−
Lacrimal gland	n/a	n/a	n/a	n/a	n/a	n/a	n/a	+	−	−
Salivary gland	−	++	+	−	n/a	++	+	++	−	−
Adrenal gland	+	+++	+	n/a	−	n/a	n/a	n/a	n/a	n/a
Lymph nodes	n/a	++	n/a	−	n/a	+	n/a	+	+	+
Tonsil	+	+	−	−	n/a	+	++	+	−	−
Spleen	−	++	−	−	−	+	++	+	−	−
Bone marrow	−	+	−	−	n/a	n/a	n/a	n/a	n/a	n/a
Heart	−	+	−	−	−	−	−	−	−	−
Skeletal muscle	−	−	−	−	−	−	−	−	−	−

+++ = high no. of positive cells; ++ = moderate no. of positive cells; + = low no. of positive cells; (+)  = questionable;

−  = no positive cells; n/a = not available.

**The nervous system** exhibited the highest number of cells positive for virus antigen. In the central nervous system (CNS), virus antigen was mainly detected in neurons, but also in glial cells (astrocytes and oligodendrocytes) (Fig. 1D). Virus antigen was present in 13/14 squirrels in all brain areas investigated, e.g. rhinencephalon, midbrain, hippocampus (Fig. 1E), cerebellum, and brain stem. In the spinal cord virus antigen was not only demonstrated in white and grey matter areas, but also in ependymal cells of the central canal. In the peripheral nervous system (PNS) virus antigen was present in nerve fibres, peri- and endoneurium of peripheral nerves of 13/14 squirrels and in neurons of the trigeminal ganglion in 6/9 squirrels and ganglia of the enteric nervous system.

**Figure 2:**
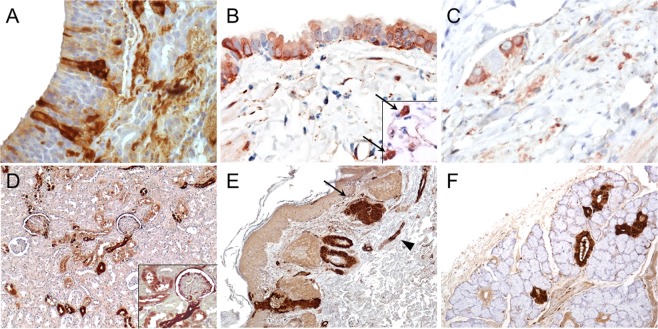
Immunohistochemistry (IHC) of different tissue sections of variegated squirrel bornavirus 1 (VSBV-1) infected variegated and Prevost’s squirrels demonstrating a wide-spread tissue distribution. (**A**) Nose of variegated squirrel V#3. Demonstration of VSBV-1 P in the olfactory epithelium; original magnification x400. (**B**) Lung, bronchus of Prevost’s squirrel P#2. VSBV-1 P in bronchial epithelium and cells with alveolar macrophage-morphology (insert, arrows); original magnification x400. (**C**) Large intestine of Prevost’s squirrel P#3. VSBV-1 P in the plexus myentericus; original magnification x400. (**D**) Kidney of variegated squirrel V#6. VSBV-1 P in a patchy distribution pattern; VSBV-1 P in Bowman capsule and podocytes of glomeruli and in tubular epithelium (insert); original magnification x100, magnification of insert x400. (**E**) Skin of Prevost’s squirrel P#7. VSBV-1 P in epithelium, sebaceous glands (arrow), hair follicles, and skin nerves (arrowhead); original magnification x100. (**F**) Lacrimal gland of Prevost’s squirrel P#8. VSBV-1 P in the epithelium of acini and intralobular ducts; original magnification x100.

In the **respiratory system**, in the nose of 10/14 animals (V#1-4, V#6, P#2, P#3, P#6-8), virus antigen was detected in olfactory and respiratory epithelial cells, glandular epithelial cells of the lamina propria, neurons, fibrocytes (Fig. 2A). In the trachea VSBV-1 P was present in 10/14 animals (V#1-3, V#5, V#6, P#1, P#2, P#6-8) in epithelial cells, connective tissue, nerves and myocytes of the lamina muscularis. In the lungs of 11/14 animals (V#1-6, P#2, P#3, P#6-8), virus antigen was found in mononuclear cells in the alveolar lumina with abundant chromatin and large nuclei, probably resembling alveolar macrophages, in type 2 pneumocytes, few bronchiolar epithelial cells, fibroblasts and smooth muscle cells (Fig. 2B).

In the **digestive system**, in all animals, except animal P#5 virus antigen was found in the pancreas, small and large intestine and stomach. In the gastrointestinal tract VSBV-1 P was present in different cell types: goblet cells, epithelial cells, smooth muscle cells, fibroblasts and in neurons of ganglia (Fig. 2C). In the pancreas virus antigen was found mainly in exocrine but also in endocrine cells. Virus antigen could not be demonstrated in the liver of any of the animals. In 6/14 animals (V#6, P#2, P#3, P#6–8) VSBV-1 P was demonstrated in glandular epithelial cells of the salivary glands and in the tunica adventitia of few vessels and in nerve fibres.

In the **urogenital system**, in kidneys of 9/14 animals (V#1, V#3, V#4, V#6, P#2, P#4, P#6-8) virus antigen was detected in epithelial cells of proximal and distal tubules, in Bowman’s capsule and cells resembling podocytes of glomeruli, typically in a patchy distribution pattern (Fig. 2D). In the urinary bladder of 8/14 animals (V#1, V#3, P#1-4, P#7, P#8) transitional epithelium, neurons and smooth muscle cells were positive. In the ovaries of 2/5 female animals (V#5, P#8) VSBV-1 P was present mainly in fibroblasts; there was no virus antigen present in the follicles. The testes of 3/9 male animals (V#4, P#2, P#6) showed immunostaining of spermatozoa and precursor cells, seminiferous tubular cells and mainly fibroblasts and artificial collagen fibres.

In the **skin** of all animals, besides Prevost’s squirrel P#5, VSBV-1 P was mainly found in the epithelium of hair follicles and sebaceous glands but also in epithelium of sweat glands, epidermis and spindle shaped cells with oval to elongated nuclei in the dermis resembling dermal nerve fibres and to a lesser extent in fibroblasts (Fig. 2E). In the **lacrimal glands** of 4/6 animals (V#2, V#4, V#6, and P#8), virus antigen was present in glandular epithelial cells, fibroblasts, nerve fibres and presumably in vascular smooth muscle cells (Fig. 2F).

In the **lymphoid system** there were only a few cells depicting virus antigen: fibroblasts and few mononuclear cells with abundant chromatin and large nuclei, probably resembling macrophages, histiocytes or dendritic cells, and few small mononuclear cells with a dark basophilic nucleus and minimal cytoplasm, resembling lymphocytes. In the tonsils of 6/14 animals (V#4, P#1, P#2, P#6-8) VSBV-1 P was demonstrated in epithelial cells. In the spleen of 5/14 animals virus antigen was found in interstitial cells, fibroblasts and mononuclear cells as described above. In the bone marrow of 1/4 animal (P#2), virus antigen was seen in few mononuclear cells and interstitial cells.

In the **heart** of 6/14 animals (V#1-4, V#6, P#2) a low number of cardiomyocytes and connective tissue harboured virus antigen whereas **skeletal muscle** cells remained negative in all animals investigated.

#### Demonstration of viral genomic and nucleoprotein (N) mRNA by in situ hybridisation

*In situ* hybridisation (ISH) was performed exemplarily on tissue sections of one Prevost’s squirrel (P#8).

In the **nervous tissue**, viral RNA was demonstrated in neurons, mainly in granular cells and pyramidal cells of the hippocampus and the cerebral cortex. A moderate number of cells exhibited mRNA whereas genomic RNA was found in a smaller number of cells regardless of the brain area investigated (Fig. 1F). In the spinal cord, viral mRNA and genomic RNA were detected in few neurons. In the trigeminal ganglion, viral mRNA was demonstrated in few ganglion cells. In peripheral nerves neither mRNA nor genomic RNA could be detected.

In the **respiratory system**, viral mRNA was present in a few cells in the lung, mostly type 2 pneumocytes, a few bronchiolar epithelial cells and mononuclear cells resembling alveolar macrophages. Neither viral genomic nor mRNA could be detected in the nose or trachea.

In the **digestive system**, viral mRNA was demonstrated in small and large intestine, respectively, genomic RNA was only found in the large intestine. In the large intestine, mRNA was found mainly in goblet cells, but also in a few enterocytes and mononuclear cells resembling dendritic cells or macrophages. Viral RNA could not be detected in ganglia or nerve fibres. In the pancreas viral RNA was present in a moderate number of pancreatic acinar cells showing an often fine granular cytoplasmic signal and to a lesser extent a nuclear signal. In the liver, oesophagus and salivary gland no viral mRNA or genomic RNA was found.

In the **urogenital system**, viral mRNA was detected in a low number of tubular epithelial cells in the kidney and transitional epithelial cells in the urinary bladder. In the ovaries neither mRNA nor genomic RNA could be detected. In the uterus viral mRNA and genomic RNA were found in a low number of glandular epithelial cells.

In the **skin**, viral mRNA and genomic RNA were identified in a few cells, mainly in epithelial cells in hair follicles and to a lesser extent in epithelial cells of the epidermis and single cells in the dermis resembling dermal nerve fibres.

In **lymphoid tissue**, viral mRNA and genomic RNA were identified in lymph nodes in a low number of cells, mainly mononuclear cells. In tonsils, spleen, lacrimal gland, heart and skeletal muscle no mRNA or genomic RNA were found.

#### Correlation of detection of virus antigen and viral RNA

The distribution of virus antigen, mRNA and genomic RNA is summarised in Table 4. Tissue distribution of virus antigen and viral RNA (RT-qPCR results from previous studies) are shown in Supplementary Table S2 with Prevost’s squirrel P#8 equating to animal identity 122/15-1^15^. Briefly, besides brain and spinal cord, peripheral organs such as large intestine, pancreas, skin and lymph nodes harbour virus antigen, genomic and mRNA as shown by IHC and ISH for P#8, which could indicate virus replication and transcription. In small intestine, lung, kidney, urinary bladder and uterus virus antigen and viral mRNA were found but no genomic virus RNA, indicating at least virus transcription. Only virus antigen but no viral RNA of any kind was demonstrated in peripheral nerves, nose, trachea, oesophagus, ovaries, eye, tonsil, spleen, salivary and lacrimal gland. In general, by ISH less cells showed a positive signal than by immunostaining by IHC in the CNS, large intestine, pancreas, kidney, uterus and skin.

## Discussion

The main objective of the study was to investigate in detail the tissue distribution of VSBV-1 in naturally infected variegated and Prevost’s squirrels. The widespread organ distribution of VSBV-1 antigen observed in this study is consistent with the assumption that both types of squirrels play a major role as virus carrier and shedder. In general, the constant presence of VSBV-1 antigen and RNA (see Supplementary Tables S1 and S2) in the central and peripheral nervous system of 13/14 animals analysed reflects the neurotropism similar to other known bornaviruses^4,15,20^. However, the widespread tissue distribution of VSBV-1 can also allow potential virus shedding via secretions and excretions such as urine, faeces, saliva and skin with subsequent virus transmission within the reservoir population as well as to accidental hosts^15^. Whether presence of virus antigen in the nose and in urogenital organs could indicate other potential transmission ways has to be further investigated. Furthermore, for VSBV-1 infection the way of virus entry, either within the potential reservoir population or for transmission to an accidental host, still is undetermined. For BoDV-1 the intranasal route of infection is assumed as the most likely way of virus entry in naturally BoDV-1-infected horses as substantiated by experimental infection of rats^21–23^. For naturally BoDV-1-infected shrews, virus antigen was also constantly found in the olfactory epithelium of the nose^8^. This would imply presence of VSBV-1 in olfactory/nasal mucosa as found here in 10 squirrels. However, for avian bornaviruses, this route does not seem to play the main role and wound/skin defects with subsequent infection of adjacent nerve fibres and viral spread to the CNS have already been documented^24,25^. It warrants further investigation which route of infection or shedding is of most importance in case of VSBV-1-infection. In principle, shedding of infectious virus can be achieved by viral presence in cells that are capable of secretion/excretion as epithelial cells which was shown for most of the organ systems, e.g. in skin, intestine and urinary bladder. An interesting finding was the simultaneous presence of viral antigen in neuroectodermal, epithelial and mesenchymal cells as already been described for neonatally BoDV-1-infected rats, naturally infected BoDV-1- infected bicolored white-toothed shrews or psittacines infected either with PaBV-2 or PaBV-4, for instance^8,10,24,26,27^. Thus, in various tissues, virus antigen staining of connective tissue (fibrocytes and collagen fibres) and smooth muscle cells have been observed similarly as for avian bornavirus infections^28,29^. However, the highest number of antigen-positive cells and consistent presence of viral mRNA and genomic viral RNA in the CNS and PNS underscore the neurotropism of VSBV-1 in naturally infected squirrel species again consistently with the findings in the above mentioned animals. In general, the tissue distribution of VSBV-1 in the investigated squirrel species closely resembles the situation in the reservoir host of BoDV-1, the bicolored white-toothed shrew with preference for the CNS as well as for secretory and excretory organs^7,8,10^. BoDV-1 antigen was constantly present in CNS and PNS with highest numbers of positive cells in the CNS^8,10^. Regular monitoring for secretion and excretion of BoDV-1 RNA in BoDV-1-infected shrews revealed that viral RNA was consistently present in swabs from saliva and skin whereas detection of RNA from lacrimal fluid, urine and faeces varied over time^8^.

Main focus of this study was to detail the dissemination of VSBV-1-antigen by IHC since previous studies already described a widespread tissue distribution of VSBV-1-RNA by RT-qPCR^15^ in naturally infected squirrels. A comparison of IHC and RT-qPCR results indicates a broad overlap of presence of virus antigen and RNA as found by IHC and ISH analyses (see Supplementary Tables S1 and S2)^15^. For instance, there was simultaneous detection of viral antigen and viral RNA by RT-qPCR detailed as mRNA or genomic RNA by ISH in brain, pancreas, skin, (uro)genital organs and mesenteric lymph nodes. Although in skeletal muscle and liver viral RNA was detected via RT-qPCR, IHC and ISH revealed negative results which might be due to a viral patchy distribution as e.g. in the kidney or low amounts of viral antigen or RNA not detectable by IHC or ISH. The detection of virus antigen or RNA in lymphoid tissues might rather represent uptake of virus products for antigen demonstration and priming of immune cells, since hemolymphatic tissue was never found to be infected by the mammalian BoDV-1. However, further investigations on the definite role of these cells have to be carried out as well as a potential entry of VSBV-1 via tonsils. There was no VSBV-1 antigen in the liver of any squirrel comparable to naturally BoDV-1- infected shrews where virus antigen was only inconsistently and viral genomic RNA was rarely found^8,10^. Histologically, in most animals a mild non-purulent meningitis and encephalitis were detected, either individually or combined, caused by infection with VSBV-1^12,15^. The lesions observed in other organs might reflect the spectrum of alterations present in squirrel populations. This comprises a considerable difference between squirrels and shrews since in BoDV-1- infected shrews no inflammatory changes in the CNS, PNS or any other organ are evident. Whether this might be due to longer evolutionary adaptation of virus and reservoir or largely depends on the time point of infection and thus immune competence remains unknown so far^7,8,10^. It has already been described that the cellular response to bornavirus infection is influenced by the age of the animal at the time point of infection and therefore also by its immune competence. In further studies it has to be addressed whether the widespread organ distribution of VSBV-1 in the squirrel reflects infection in a state of immunotolerance as shown for neonatally BoDV-1-infected rats. No clinical signs were observed in shrews or squirrels, regardless of presence of inflammatory lesion.

In summary, the disseminated virus spread in naturally VSBV-1-infected squirrels seems to be essential to ensure virus maintenance in the reservoir population but represents an important risk factor for spill over and infection of accidental hosts, a scenario which occurs more frequently in RNA-viruses with a higher host plasticity^30^. Further research on the infection cycle of VSBV-1, the reservoir role of squirrel species and routes of transmission to humans is urgently needed for a reliable risk assessment, prevention strategies and treatment options.

## Material and Methods

Six naturally VSBV-1-infected adult variegated squirrels V#1 to V#6 (4 males, 2 females) and eight naturally VSBV-1 infected adult Prevost’s squirrels P#1 to P#8 (5 males, 3 females) from different breeding colonies in Germany (9 animals) and the Netherlands (5 animals) were available for investigation^14,15^. All six variegated and seven Prevost’s squirrels were euthanized by veterinarians for health risk assessment and management after being tested positive for VSBV-1 RNA in oropharyngeal swabs to prevent further distribution in the squirrel population and possible virus transmission to humans. One of the Prevost’s squirrels (P#5, male) died before testing probably due to a trauma and was frozen in −20 °C until necropsy. This animal was also tested positive for VSBV-1 RNA by PCR. All animals were kept in private holdings and no experiments were performed on live animals. Post mortem examination was performed to identify gross and microscopic lesions. Representative organ samples listed in Tables 3 and 4 were fixed in 10% buffered formalin for 21 days, that was renewed once after 24 hours and embedded in paraffin.

### Immunohistology

Immunohistochemistry (IHC) was performed on formalin-fixed, paraffin-embedded (FFPE) tissue by using the polyclonal rabbit anti-BoDV-P (p24) antibody directed against the bornaviral phosphoprotein (P) with proven cross-reactivity against VSBV-1 and other bornaviruses as described elsewhere^12,31–33^. Tissue of a naturally BoDV-1-infected horse and a VSBV-1-negative squirrel served as positive and negative control. Additionally, negative controls for each slide consisted of omitting the primary antibody and using a normal rabbit control serum instead.

### *In situ* hybridisation

*In situ* hybridisation (ISH) was performed on Prevost’s squirrel P#8 as an example to identify genomic viral RNA and mRNA sequences encoding for VSBV-1 nucleoprotein (N) following protocols described elsewhere^34^. This N gene region was chosen for ISH due to the known abundant protein expression in BoDV-1 and in parrot bornavirus (PaBV)-infections^34–38^. Specific RNA probes were designed, and PCR was performed using two primers with a length of about 130 nucleotides, with an overlapping region of 30 nucleotides at the 3′-terminal region and corresponding 20 bp long primers at both 5′-terminal regions, generating a probe of 230 nucleotides equating to position 100–330 of the VSBV-1 genome VSBV-1/BH55/16 [GenBank MF597762.1]. The PCR-product was ligated into a pCR™4-TOPO® TA-vector (TOPO® TA Cloning® Kit, ThermoFisher) and reamplified using manufacturer’s M13 forward and M13 reverse primers combined with VSBV-1 specific primers. Two RNA probes detecting sense and antisense RNA were generated according to manufacturer’s guide (DIG RNA Labeling Kit, Sigma-Aldrich).

### Evaluation of IHC and ISH

IHC- and ISH-analysis were performed applying a semi-quantitative scoring system depicted in Table 5. To address differences between staining patterns in various tissues a two-step analysis was needed. Initially, the percentage of positive cells in a given slide was graded in 40x magnification and subsequently the average number of positive cells in five high power fields (HPFs) with the highest number of positive cells was evaluated. The final score represents the mean of both scores.

**Table 5:** Scoring system for immunohistochemistry and *in situ* hybridization.

Score	Overview in 40x magnification in %	Overview in 400x magnification, mean of cells in 5 HPFs*
−	0	0
+	1–15	1–30
++	15–40	30–80
+++	40–65	80–150
++++	>65	>150

HPF = high power field.

## Supplementary information

Correlation between immunohistochemical (IHC) evaluation of tissues from VSBV-1-infected squirrels and RT-qPCR-results

## Data Availability

All data generated or analysed during this study are included in this published article.
