# Supplementary material for: Distribution of zoonotic variegated squirrel bornavirus 1 in naturally infected variegated and Prevost’s squirrels
Source: Sci Rep. 2019 Aug 6;9:11402. doi: 10.1038/s41598-019-47767-4 (PMC6684602; doi:10.1038/s41598-019-47767-4)
Supplement: Supplementary file 1 — Correlation between immunohistochemical (IHC) evaluation of tissues from VSBV-1-infected squirrels and RT-qPCR-results [file 41598_2019_47767_MOESM1_ESM.docx]

**Distribution of zoonotic variegated squirrel bornavirus 1 in naturally infected variegated and Prevost´s squirrels**

Jana Petzold^+^, Judith M.A. van den Brand^+^, Daniel Nobach, Bernd Hoffmann, Donata Hoffmann, Christine Fast, Chantal B.E.M Reusken, Peter R.W.A van Run, Kore Schlottau, Martin Beer, and Christiane Herden

^+^These first authors contributed equally to this article.

Supplementary Table S1. Correlation between immunohistochemical (IHC) evaluation of tissues from VSBV-1-infected variegated squirrels (shown in table 3 in this manuscript) and RT-qPCR-results from previous studies ^15^.

|  | V#1  *48/15-1^15^ | | V#2  *48/15-2^15^ | | V#3  *48/15-11^15^ | | V#4  *49/15-1^15^ | | V#5  *49/15-6^15^ | | V#6  *75/15^15^ | |
| --- | --- | --- | --- | --- | --- | --- | --- | --- | --- | --- | --- | --- |
|  | IHC | RT-qPCR^15^ | IHC | RT-qPCR^15^ | IHC | RT-qPCR^15^ | IHC | RT-qPCR^15^ | IHC | RT-qPCR^15^ | IHC | RT-qPCR^15^ |
|  |  |  |  |  |  |  |  |  |  |  |  |  |
| Brain | ++ | +++ | + | +++ | ++ | +++ | +(+) | +++ | ++ | +++ | ++ | +++ |
| Spinal cord/ medulla oblongata | ++ | +++ | +++ | +++ | +++ | +++ | ++ | +++ | ++ | +++ | +++ | +++ |
| Peripheral nerves | ++ | ++ | ++ | ++ | ++(+) | ++ | ++ | ++ | ++ | ++ | +++ | ++ |
| Nose | ++ | ++ | + | ++ | +++ | +++ | ++ | +++ | - | ++ | +++ | +++ |
| Trachea | + | n/a | + | n/a | + | n/a | n/a | n/a | + | n/a | ++ | n/a |
| Lung | + | ++ | + | ++ | + | +++ | + | +++ | + | ++ | ++ | ++ |
| Oesophagus | ++ | n/a | + | n/a | ++ | n/a | + | n/a | + | n/a | + | n/a |
| Stomach | + | n/a | + | n/a | n/a | n/a | n/a | n/a | n/a | n/a | n/a | n/a |
| Small intestine | - | n/a | + | n/a | + | n/a | n/a | n/a | - | n/a | + | n/a |
| Large intestine | + | n/a | +(+) | n/a | +(+) | n/a | + | n/a | ++ | n/a | - | n/a |
| Liver | - | ± | - | ± | - | + | - | + | - | ++ | - | + |
| Pancreas | + | ++ | + | +++ | ++ | ++ | ++ | ++ | + | ++ | ++ | ++ |
| Kidney | + | ++ | - | + | + | ++ | +++ | +++ | - | +++ | ++ | ++ |
| Urinary bladder | ++ | ++ | n/a | ++ | ++ | +++ | - | +++ | - | ++ | n/a | + |
| Ovary | n/a | n/a | n/a | +++ | n/a | n/a | n/a | n/a | + | + | n/a | n/a |
| Uterus | n/a |  | + |  | n/a |  | n/a |  | - |  | n/a |  |
| Testis | - | + | n/a | n/a | - | + | + | +++ | n/a | n/a | - | +++ |
| Penis | - |  | n/a |  | + |  | - |  | n/a |  | + |  |
| Skin | + | ++ | + | + | + | ++ | ++ | ++ | + | ++ | + | ++ |
| Eye | + | n/a | + | n/a | + | n/a | n/a | n/a | + | n/a | ++ | n/a |
| Lacrimal gland | - | n/a | + | n/a | n/a | n/a | + | n/a | - | n/a | + | n/a |
| Eye swab sample | n/a | + | n/a | ± | n/a | + | n/a | + | n/a | ± | n/a | + |
| Salivary gland | - | ++ | - | + | - | + | n/a | ++ | - | ++ | + | ++ |
| Adrenal gland | n/a | n/a | n/a | n/a | n/a | n/a | n/a | n/a | n/a | n/a | ++ | n/a |
| Lymph nodes | - | ++ | n/a | ++ | - | +++ | - | ++ | - | ++ | n/a | + |
| Tonsil | n/a | ++ | n/a | ++ | n/a | ++ | + | ++ | - | + | n/a | ++ |
| Spleen | - | + | - | ± | - | ++ | - | ++ | - | + | + | ++ |
| Heart | + | ++ | + | + | + | ++ | + | ++ | - | ++ | + | ++ |
| Skeletal muscle | - | + | - | ± | - | + | - | ++ | - | + | - | ++ |
|  |  | |  | |  | |  | |  | |  | |
| IHC: +++ = high no. of positive cells; ++ = moderate no. of positive cells; + = low no. of positive cells; (+) = questionable;  - = no positive cells; n/a = not available;  RT-qPCR, data are represented in VSBV-1 genome equivalent copies per millilitre of template: +++ = >10^6^; ++ = 10^4^-10^6^; + = 10^2^-10^4^; ± = 10^0^-10^2^; n/a = not available; data are published in previous studies^15^  * Animal number in previous studies^15^ equating to animal number V#(x) | | | | | | | | | | | | |

Supplementary Table S2. Correlation between immunohistochemical (IHC) evaluation of tissues from VSBV-1-infected Prevost´s squirrels (shown in table 4 in this manuscript) and RT-qPCR-results from previous studies ^15^.

|  | P#1  *3/16-1^15^ | | P#2  *3/16-2^15^ | | P#3 | | P#4 | | P#5 | | P#6  *133/15^15^ | | P#7  *122/15-2^15^ | | P#8  *122/15-1^15^ | |
| --- | --- | --- | --- | --- | --- | --- | --- | --- | --- | --- | --- | --- | --- | --- | --- | --- |
|  | IHC | RT-qPCR^15^ | IHC | RT-qPCR^15^ | IHC | RT-qPCR | IHC | RT-qPCR | IHC | RT-qPCR | IHC | RT-qPCR^15^ | IHC | RT-qPCR^15^ | IHC | RT-qPCR^15^ |
|  |  |  |  |  |  |  |  |  |  |  |  |  |  |  |  |  |
| Brain | + | +++ | + | +++ | + | ++ | + | ++ | - | ++ | +++ | +++ | +++ | +++ | +++ | ++ |
| Spinal cord/ medulla oblongata | ++ | +++ | ++ | +++ | n/a | ++ | (+) | ++ | n/a | n/a | ++ | ++ | +++ | ++ | +++ | ++ |
| Peripheral nerves | + | + | + | ++ | (+) | n/a | (+) | n/a | n/a | n/a | ++ | ++ | + | ++ | +++ | ++ |
| Nose | - | ++ | +++ | +++ | + | n/a | - | n/a | n/a | n/a | ++ | ++ | ++ | ++ | + | + |
| Trachea | + | n/a | ++ | n/a | - | n/a | - | n/a | - | n/a | + | n/a | + | n/a | + | n/a |
| Lung | - | ± | + | ++ | (+) | + | - | ++ | - | ++ | + | ++ | + | + | + | ++ |
| Oesophagus | n/a | n/a | n/a | n/a | + | n/a | - | n/a | - | n/a | + | n/a | + | n/a | + | n/a |
| Stomach | ++ | n/a | ++ | n/a | + | n/a | - | n/a | - | n/a | n/a | n/a | n/a | n/a | n/a | n/a |
| Small intestine | + | n/a | ++ | n/a | + | +++ | + | +++ | - | ++ | ++ | n/a | + | n/a | + | n/a |
| Large intestine | ++ | n/a | + | n/a | + | +++ | - | +++ | - | +++ | + | n/a | +++ | n/a | ++ | n/a |
| Liver | - | ± | - | + | - | + | - | + | - | + | - | + | - | + | - | + |
| Pancreas | +++ | + | ++ | ++ | + | + | + | ++ | - | +++ | ++ | ++ | + | ++ | +++ | ++ |
| Kidney | - | ++ | ++ | +++ | - | ± | + | + | - | ++ | ++ | ++ | ++ | ++ | ++ | ++ |
| Urinary bladder | + | ++ | ++ | ++ | + | n/a | + | n/a | - | n/a | n/a | ++ | + | ++ | ++ | ++ |
| Ovary | - | ++ | n/a | n/a | - | + | n/a | n/a | n/a | n/a | n/a | n/a | n/a | n/a | ++ | +++ |
| Uterus | + |  | n/a |  | - |  | n/a |  | n/a |  | n/a |  | n/a |  | ++ |  |
| Testis | n/a | n/a | + | +++ | n/a | n/a | - | + | - | + | + | ++ | - | ++ | n/a | n/a |
| Penis | n/a |  | - |  | n/a |  | n/a |  | n/a |  | + |  | - |  | n/a |  |
| Skin | + | + | + | ++ | + | + | + | ++ | - | + | ++ | ++ | ++ | ++ | ++ | ++ |
| Eye | + | n/a | + | n/a | + | n/a | + | n/a | n/a | n/a | ++ | n/a | +++ | n/a | ++ | n/a |
| Lacrimal gland | n/a | n/a | n/a | n/a | n/a | n/a | n/a | n/a | n/a | n/a | n/a | n/a | n/a | n/a | + | n/a |
| Eye swab sample | n/a | n/a | n/a | n/a | n/a | n/a | n/a | n/a | n/a | n/a | n/a | ± | n/a | ± | n/a | n/a |
| Salivary gland | - | ± | ++ | +++ | + | ++ | - | ± | n/a | + | ++ | ++ | + | ++ | ++ | ++ |
| Adrenal gland | + | n/a | +++ | n/a | + | ++ | n/a | ++ | - | +++ | n/a | n/a | n/a | n/a | n/a | n/a |
| Lymph nodes | n/a | + | + | ++ | n/a | + | - | ± | n/a | + | + | ++ | n/a | ++ | + | ++ |
| Tonsil | + | +++ | + | ++ | - | ++ | - | ++ | n/a | + | + | ++ | ++ | ++ | + | ++ |
| Spleen | - | ± | ++ | +++ | - | + | - | ± | - | ++ | + | ++ | ++ | ++ | + | ++ |
| Heart | - | + | + | ++ | - | ++ | - | + | - | ++ | - | ++ | - | + | - | + |
| Skeletal muscle | - | + | - | ++ | - | + | - | + | - | ± | - | ++ | - | ++ | - | ++ |
|  |  | |  | |  | |  | |  | |  | |  | |  | |
| IHC: +++ = high no. of positive cells; ++ = moderate no. of positive cells; + = low no. of positive cells; (+) = questionable; - = no positive cells; n/a = not available;  RT-qPCR, data are represented in VSBV-1 genome equivalent copies per millilitre of template: +++ = >10^6^; ++ = 10^4^-10^6^; + = 10^2^-10^4^; ± = 10^0^-10^2^; n/a = not available; data are published in previous studies for P#1, P#2, P#6-8^15^  * Animal number in previous studies^15^ equating to animal number V#(x) | | | | | | | | | | | | | | | | |
